# Pulmonary Embolism Presenting As Shoulder and Back Pain: A Case Report

**DOI:** 10.7759/cureus.64016

**Published:** 2024-07-07

**Authors:** Chukwuemeka Nwaneri, Rebecca Race, Romoluwa Oladele, Subramanian Kumaran

**Affiliations:** 1 Emergency Medicine Department, Shrewsbury and Telford NHS Trust, The Royal Shrewsbury Hospital, Shrewsbury, GBR; 2 Emergency Medicine Department, The Shrewsbury and Telford Hospital NHS Trust, The Royal Shrewsbury Hospital, Shrewsbury, GBR

**Keywords:** prophylactic and therapeutic anticoagulation, shortness of breath (sob), shoulder tip pain, atypical chest pain, acute pulmonary embolism

## Abstract

Pulmonary embolism (PE) is a common but life-threatening condition, and diagnosis can be challenging. Diagnosis is even more difficult in those patients with atypical presentations such as the absence of pleuritic chest pain, dyspnoea, tachycardia, or symptoms of deep vein thrombosis. We have delineated shoulder and back pain as an atypical sign of PE. However, the significant amount of misdiagnosis highlights the importance of other rare symptoms of this potentially fatal disease. Therefore, eliciting these rare presenting symptoms can significantly reduce morbidity and mortality. Here, we report the case of a patient who, 13 days after a laparoscopic Nissen fundoplication, presented to the emergency department (ED) with left shoulder and left-sided pleuritic back pain. She was managed in the resuscitation area in the ED and was subsequently diagnosed with a left-sided PE. Her care was taken over by the medical team, and she continued her recovery in the acute medical unit.

## Introduction

Pulmonary embolism (PE) is an obstruction within the pulmonary vasculature caused by blood clots, air, tumors, or fat. It is a serious, life-threatening medical condition, particularly when early identification, diagnosis, and treatments are not instituted. The hallmark symptoms are pleuritic chest pain, dyspnea, tachycardia, cough, hemoptysis, and symptoms of deep vein thrombosis (DVT). Syncope or hypotension (obstructive shock) results from three common underlying pathogeneses: pulmonary infarction; acute, unexplained shortness of breath without pulmonary infarction; and right ventricular failure [1,2]. The major cause of death in severe PE is right ventricular failure due to acute pressure overload.

Other uncommon symptoms documented in the literature include abdominal or flank pain, drenching night sweats, retrosternal non-pleuritic chest pain, confusion, seizures, bradycardia, syncope, and cardiac arrest [3-6]. Lee et al. documented neck-to-shoulder referred pain as an unusual presentation of PE in a patient with cervical spinal cord injury [7].

Pulmonary embolism is the third most common cause of death from cardiovascular disease after myocardial infarction and cerebrovascular accident [8]. Globally, the incidence of PE is 120 per 100,000 cases as of 2016 [9]. However, in the United Kingdom, the annual incidence is 30 to 40 per 100,000 people [10]. The mortality rate of PE is 30% without treatment [3,11]. Invariably, where patients have high-risk PE associated with cardiovascular instability, the risk of death has been quoted as more than 50% [12]. Statistics from post-mortem studies show that 70% of PEs have been misdiagnosed [13]. In this case report, we describe an uncommon presentation of PE, in which clinical suspicion was initially low due to atypical symptoms, i.e., shoulder-to-back pain.

## Case presentation

A 70-year-old lady presented to the ED with a 12-hour history of worsening atraumatic left shoulder and back pain. She had struggled to control her pain with regular analgesia for three days before presentation. The numerical pain severity scale rating was 10 out of 10, sharp in nature, and non-radiating. However, initially, pain was relieved by analgesia but became increasingly worse despite taking maximum doses. She did not give any history suggestive of pleuritic chest pain, cough, shortness of breath, hemoptysis, fever, or lower limb swelling or pain.

Two weeks before this presentation, she underwent laparoscopic Nissen’s fundoplication for a paraesophageal hiatus hernia. Her recovery was uneventful, and she was discharged on day two post-operatively with analgesia and prophylactic doses of low molecular weight heparin (LMWH) anticoagulant for seven days. Her past medical history included ischemic heart disease and gastric outlet reflux disease, and her medications were amitriptyline and omeprazole.

On examination, she was conscious, and alert, with a Glasgow coma scale (GCS) of 15 out of 15, anicteric, not pale, not dehydrated, acyanosed, and afebrile at 37.4oC. However, she was in some distress and was obviously uncomfortable and unable to lie still on the trolley. There was no significant pedal edema and no calf swelling or tenderness on palpation. Her blood pressure was elevated at 165 mmHg systolic and 95 mmHg diastolic. She had a good volume and regular tachycardia of 102 bpm. Jugular venous pressure was not elevated, and she had no added heart sounds.

Her respiratory rate was normal, and her oxygen saturation was 98% on room air. Auscultation of her lung fields revealed equal air entry bilaterally with normal breath sounds throughout. Abdominal examination was unremarkable. The surgical wounds were intact and healing well, with no signs of inflammation or infection.

The left shoulder had a normal contour with no evidence of any deformity or swelling, and there was a full range of movement. Her left posterior chest wall showed no evidence of swelling, abrasions, or bruising but was diffusely tender on deep palpation. Her blood results revealed some abnormalities at the time of presentation (Table 1).

**Table 1:** Blood results with reference ranges

Variable	Value	Normal reference range
White blood cell count	15.0 X 10^9^/L	4.0 - 11.0 X 10^9^/L
Neutrophil count	11.9 X 10^9^/L	1.5 - 8.0 10^9^/L
C-reactive protein	58mg/L	0-5mg/L
D-dimer	1321µg/L	<500µg/L

**Figure 1:**
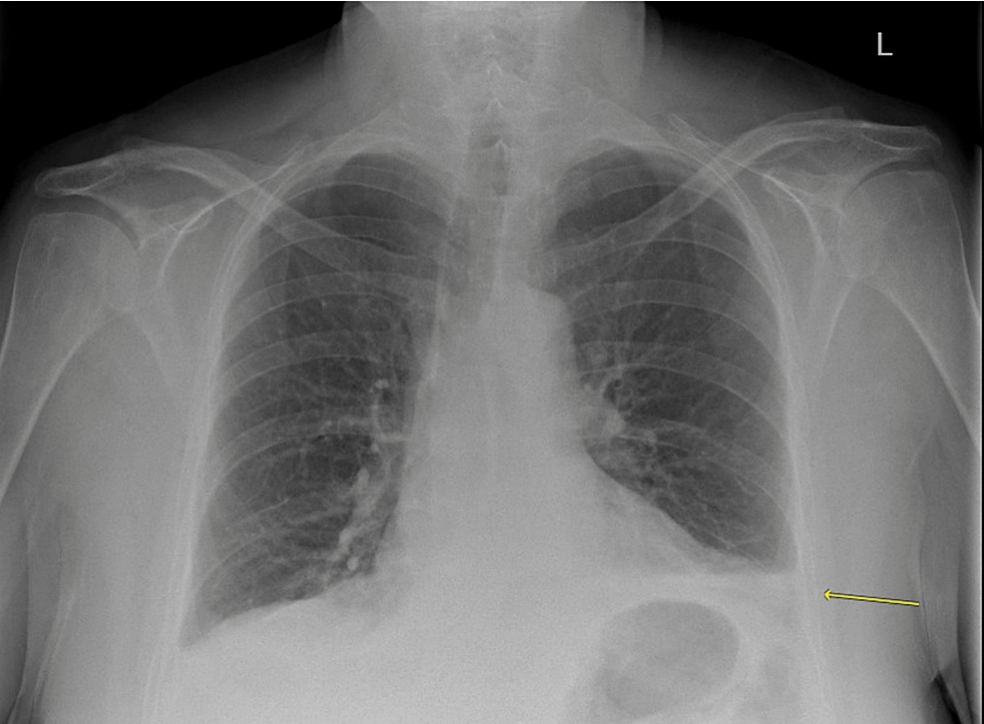
Chest X-ray showing left-sided pleural effusion, with an area of left lower lobe consolidation (yellow arrow) suggestive of Hampton’s hump infarct. The left lower lobe also shows regional oligaemia distal to the occluded vessel (consistent with Westermark sign suggestive of PE). PE: Pulmonary embolism

The white blood cell count, neutrophil count, C-reactive protein (CRP), and D-dimer were all increased. However, her remaining blood tests were within normal limits. The electrocardiogram (ECG) tracing showed sinus tachycardia. Her chest X-ray revealed atelectasis in the left base of the lung, with a loss of left costophrenic angle indicating small pleural effusion. However, there was no evidence of a pneumothorax or gas under the diaphragm (Figure 1).

**Figure 2:**
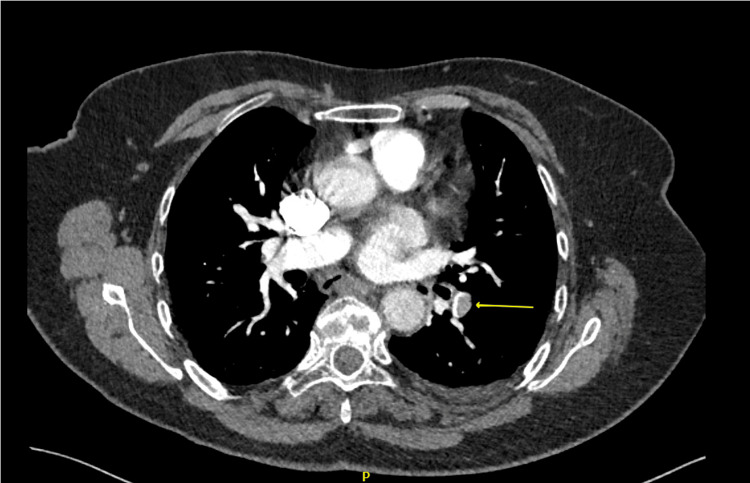
The CTPA showing filling defects in the left pulmonary artery (pointing yellow arrow), suggestive of left-sided PE. CTPA: Computed tomography pulmonary angiography, PE: Pulmonary embolism

Initially, the patient’s presentation was attributable to a lower respiratory tract infection and/or pneumonia. However, a computed tomographic pulmonary angiogram (CTPA) showed pulmonary emboli in the distal end of the left pulmonary artery extending into the lower lobe branches. These findings are in keeping with right heart strain, left lower lobe consolidation, bilateral small pleural effusions, as well as collapsed left lower lung parenchyma due to lung infarction (Figure 2).

Analgesia was optimized using intravenous morphine, and she started a therapeutic dose of subcutaneous LMWH as well as intravenous antibiotics as per the local protocols. However, she later developed shortness of breath and left-sided chest pain and required supplemental oxygen of 2L/min to maintain her oxygen saturation between 94% and 98%.

The patient's clinical state improved, and she was weaned off her oxygen therapy and was discharged home to continue her non-vitamin K antagonist oral anticoagulant (NOAC) for three months as per current guidelines for provoked PE, as well as a seven-day course of oral antibiotics. The NOACs are often preferred over warfarin due to their predictable pharmacokinetics, fewer dietary restrictions and drug-drug interactions, and less frequent need for monitoring.

## Discussion

Clinical presentation of PE can be asymptomatic but can also present with hallmark features of shortness of breath, pleuritic chest pain, cough, hemoptysis, tachycardia, or tachypnoea that vary from mild PE (small embolus with low mortality) through massive PE with life-threatening presentations, resulting from right ventricular failure, shock, and/or death.

Initially, the differential diagnoses included perforated bowel (recent abdominal surgery), pneumothorax, undiagnosed lung cancer, neuropathic pain, myofascial pain syndrome, rotator cuff syndrome, pneumonia, myocardial infarction, or dissection of the aorta. However, since the shoulder and back examination both appeared unremarkable, we surmised that the pain was more likely to originate from the viscera (left lower lobe PE) and had been perceived as somatic pain (referred pain to the back and shoulder). Ruch and Patton used the convergence-projection theory to explain the possible cause of the referred pain. They stressed that visceral and somatic afferent fibers converge in the common dorsal horn neuron, and upon activation of pain receptors in the visceral organ, there is a concomitant perception of pain in the somatic nerve distribution since the central nervous system is unable to differentiate the origin of pain [14]. Back pain as a symptom of PE could therefore reflect posterior pulmonary infarction. Lung infarction following distal pulmonary artery occlusion is most commonly caused by acute PE. In addition, lung infarction can cause pleural damage, leading to inflammation, which in turn causes pain. Referred shoulder pain can be so severe that the patient may have difficulty lying down or sleeping in a recumbent position, as seen in our patient [15,16].

Reports have shown that some factors specific to laparoscopic surgery may increase the risk of DVT and PE. These factors include carbon dioxide pneumoperitoneum, reverse Trendelenburg position, prolonged operation time, and immobilization [17-19]. High-mortality patients may also present with syncope, myocardial infarction, cardiac arrhythmias, shock, hemodynamic instability, and cardiac arrest predominantly from asystole.

Atypical presentation may delay the diagnosis of PE, and patients receiving anticoagulant medication may provide false reassurance. The patient described in this report had received a prophylactic dose of LMWH during her hospital admission and NOAC for one week at home and still developed a PE. This is often described as anticoagulation failure [20].

Further diagnostic delays can be seen in patients whose clinical presentations coexist with a septic picture with or without pneumonia, as seen in our patient. It is worth noting that the absence of shortness of breath, pleuritic chest pain, or cough should not be used to rule out PE; rather, a high index of suspicion should be instituted in those patients at high risk of developing PE.

Raised D-dimer is notoriously non-specific and may also be seen in other conditions, particularly after surgery and in patients placed on prophylactic doses of anticoagulants. The ECG revealed sinus tachycardia, which can be seen in both pneumonia and PE. Chest X-ray showed left-sided pleural effusion with an area of left lower lobe consolidation (suggestive of Hampton’s hump infarct). The left lower lobe also showed regional oligaemia distal to the occlusion (consistent with the Westermark sign suggestive of PE). Despite these recognized signs, the gold standard is CTPA, which in this case showed a filling defect in the left pulmonary artery, which confirmed a left-sided PE. As a result of the CTPA findings, diagnosis was prompt and appropriate management was initiated. Therapeutic anticoagulation was instituted and remains the treatment modality of choice for patients with PE. Our patient was started on a therapeutic dose of LMWH and then discharged on NOAC for three months as per current guidelines for provoked PE [20].

## Conclusions

Shoulder and back pain are uncommon symptoms of acute PE. This case report highlights an unusual presentation of PE in an elderly woman who presented with atypical symptoms of severe atraumatic left shoulder and back pain. However, since PE is a potentially acute life-threatening condition, a high index of suspicion from the outset and a well-timed diagnosis and management plan are paramount to decreasing mortality. Therapeutic anticoagulation remains the cornerstone of PE treatment, as demonstrated in the successful management of this patient's condition.
